# Effects of early feeding on growth velocity and overweight/obesity in a cohort of HIV unexposed South African infants and children

**DOI:** 10.1186/s13006-015-0041-x

**Published:** 2015-04-02

**Authors:** Vundli Ramokolo, Carl Lombard, Meera Chhagan, Ingunn MS Engebretsen, Tanya Doherty, Ameena E Goga, Lars Thore Fadnes, Wanga Zembe, Debra J Jackson, Jan Van den Broeck

**Affiliations:** Health Systems Research Unit, South African Medical Research Council, Cape Town, South Africa; Centre for International Health, Department of Global Public Health and Primary Care, University of Bergen, Bergen, Norway; Biostatistics Unit, South African Medical Research Council, Cape Town, South Africa; School of Public Health, University of the Western Cape, Cape Town, South Africa; Department of Pediatrics, University of KwaZulu Natal, KwaZulu Natal, South Africa; Department of Clinical Dentistry, University of Bergen, Bergen, Norway; Department of Paediatrics and Child Health, Kalafong Hospital, University of Pretoria, Pretoria, South Africa

## Abstract

**Background:**

South Africa has the highest prevalence of overweight/obesity in Sub-Saharan Africa. Assessing the effect of modifiable factors such as early infant feeding on growth velocity and overweight/obesity is therefore important. This paper aimed to assess the effect of infant feeding in the transitional period (12 weeks) on 12–24 week growth velocity amongst HIV unexposed children using WHO growth velocity standards and on the age and sex adjusted body mass index (BMI) Z-score distribution at 2 years.

**Methods:**

Data were from 3 sites in South Africa participating in the PROMISE-EBF trial. We calculated growth velocity Z-scores using the WHO growth standards and assessed feeding practices using 24-hour and 7-day recall data. We used quantile regression to study the associations between 12 week infant feeding and 12–24 week weight velocity (WVZ) with BMI-for-age Z-score at 2 years. We included the internal sample quantiles (70th and 90th centiles) that approximated the reference cut-offs of +2 (corresponding to overweight) and +3 (corresponding to obesity) of the 2 year BMI-for-age Z-scores.

**Results:**

At the 2-year visit, 641 children were analysed (median age 22 months, IQR: 17–26 months). Thirty percent were overweight while 8.7% were obese. Children not breastfed at 12 weeks had higher 12–24 week mean WVZ and were more overweight and obese at 2 years. In the quantile regression, children not breastfed at 12 weeks had a 0.37 (95% CI 0.07, 0.66) increment in BMI-for-age Z-score at the 50th sample quantile compared to breast-fed children. This difference in BMI-for-age Z-score increased to 0.46 (95% CI 0.18, 0.74) at the 70th quantile and 0.68 (95% CI 0.41, 0.94) at the 90th quantile . The 12–24 week WVZ had a uniform independent effect across the same quantiles.

**Conclusions:**

This study demonstrates that the first 6 months of life is a critical period in the development of childhood overweight and obesity. Interventions targeted at modifiable factors such as early infant feeding practices may reduce the risks of rapid weight gain and subsequent childhood overweight/obesity.

**Electronic supplementary material:**

The online version of this article (doi:10.1186/s13006-015-0041-x) contains supplementary material, which is available to authorized users.

## Introduction

The first 1000 days from conception to 2 years are a critical period in the growth and development of infants [1]. Insults or stimuli in the intrauterine environment, including maternal body composition and diet, can “programme” the expression of genes and lead to permanent physiological or morphological changes in the fetus [2-4]. Programming can extend into the postnatal period where infant feeding and infant growth patterns can further predispose infants to later cardiovascular disease, overweight and obesity and other chronic disease; a phenomenon called “developmental origins of health and disease”[2,5]. Evidence from twin studies suggests that rapid weight gain between birth and 3 months is primarily influenced by modifiable environmental influences in term babies [6-13], such as nutrition [14], whereas from 5 months onwards genetic factors play a larger role [7,9,15].

The World Health Organisation (WHO) recommends exclusive breastfeeding (EBF) until 6 months of age and timely introduction of appropriate complementary foods with continued breastfeeding up to 2 years or beyond [16]. EBF protects infants from rapid weight gain in the postnatal period, a risk factor for insulin resistance and later overweight and obesity which are both prevalent in South Africa [17,18]. Breastfeeding practices are still poor in South Africa as only 8% of infants 0–5 months are EBF [19]. We have shown that a third of infants who initiated breastfeeding in our sample were introduced to other foods, particularly formula, from as early as 3 days [20]. By 12 weeks postpartum 20% and 40% of HIV negative and positive women respectively had stopped all breastfeeding and transitioned to other liquids and solids Data from observational studies showed that children introduced to complementary foods earlier than 3 or 4 months, compared with later introduction, were more likely to be overweight or obese [21-24]. A randomised controlled trial also showed that high protein intake in infant formula was associated with rapid weight gain in the first 2 years of life [25]. Here we aimed to assess the effect of infant feeding in the transitional period (12 weeks) on 12–24 week growth velocity amongst HIV unexposed children using WHO growth velocity standards [26], which can adequately describe the growth of South African children [27], and on the age and sex adjusted body mass index (BMI) Z-score distribution at 2 years. We used data from the PROMISE-EBF (ClicinalTrials.gov.no: NCT00397150) trial; a multi-country cluster randomized community trial primarily assessing the effect of home based EBF counselling on EBF rates at 12 weeks [28].

## Methods

### Study design and participants

The present paper includes data from the three South African sites of the PROMISE-EBF behavioural-intervention trial that sought to improve EBF rates through peer counselling, conducted between 2006 and January 2008: Paarl (mixed peri-urban/rural area), Rietvlei (rural area) and Umlazi (peri-urban formal township). Trial methods have been described in detail elsewhere [28,29]. Briefly, pregnant women in their last trimester of pregnancy were screened for inclusion into the study. A total of 964 HIV negative and 184 HIV positive women and their singleton children were enrolled at the 3 week postnatal visit and followed up at 6, 12 and 24 weeks . Six hundred and fifty four HIV unexposed children (67.8% of original cohort) followed up again between March and September 2008 at a median age of 22 months (IQR: 9–34 months), which we refer to as the 2 year visit, were considered for this analysis due to the described negative effect of HIV infection on growth [27]. We compared baseline characteristics of participants that were followed-up at 2 years with those that were not (see Additional file 1) and observed no systematic differences, besides the proportion of male children, between the groups. This suggests that the sample that was followed-up is generally representative of the children in the whole cohort. A further 13 children were excluded because of extreme and implausible anthropometric values leading to a final sample of 641 children.

### Data collection

Standardised questionnaires were used to collect interview data during pregnancy and postnatally at 3, 6, 12 and 24 weeks, and at the primary endpoint of 2 years of age. Maternal variables included: age, parity and education which were captured during recruitment; delivery mode and reported HIV status collected at the 3 week visit. The questionnaires also addressed infant feeding practices through 24-hour and 7-day recall of a list of 23 foods commonly consumed in the study sites. No food diaries were used. Data on child birth weight were extracted from perinatal records.

Field staff measured child weight and recumbent length/height during the 3, 6, 12, and 24 weeks visits and at 2 years. Children were weighed to the nearest 0.1 kg on Masskot (SOS Series) electronic pan scales*,* which were calibrated weekly using a 2 kg weight, wearing minimum clothing and no shoes*.* Depending on the study site, recumbent length measurements were obtained to the nearest 0.1 cm using TALC roller meters (Oxford, UK) or Shorr Height-Length Measuring Board (Maryland, USA) while height was measured using a validated ustom-made stadiometer. All field workers were trained on anthropometric techniques. In order to improve validity and reduce inter and intra-observer bias, the anthropometry data collection was validated periodically. Child age was calculated using the date of birth from the Road to Health card and the date of the interview.

Data were double-entered into a Microsoft Access database and analysed using Stata SE 12 [30] and IBM SPSS Statistics 21 [31].

### Anthropometric scoring

The primary outcome measure was BMI-for-age Z-scores at 2 years; secondary outcomes were weight velocity Z-scores (WVZ) and length velocity Z-scores (LVZ). We calculated BMI-for-age Z-scores at the 12 week and 2 year visits, standardised for sex and actual age at the respective visit, using the WHO growth standards [32]. We considered children as “overweight” and “obese” if their BMI-for-age Z-scores were above +2 and +3 respectively as recommended by the World Health Organisation [33]. A macro based on the WHO-2009 growth velocity standards was used to compute the WVZ and LVZ. Velocities were calculated for a first period, namely from 3 or 6 to 12 weeks post-delivery, and for a second period, namely 12 to 24 weeks post-delivery. In cases where the 3 or 6 week weight was missing we used the birth weight for the calculation of velocity in the first period. The age intervals and child ages observed in the study did not always correspond exactly with those of the velocity standards. Thus the velocity Z-scores were calculated, as recommended by WHO, by identifying the best-fitting age interval for each child period observed and linearly extrapolating the observed increment in the child period to the duration of the best-fitting target interval [26].

### Data cleaning

Anthropometric measurement values and Z-scores were flagged for verification if any of the following criteria were met: a) decrease in length of more than 2 cm between two consecutive visits; b) WAZ <-6 or >5, WLZ <-5 or >5, LAZ <-6 or >6, WLZ >3 and LAZ <-3; c) extreme changes in LAZ between visits defined as LAZ at 3 weeks < 2 and LAZ at 24 weeks > 2.5, or LAZ at 24 weeks < 2 and LAZ at 36 weeks > 2.5; d) changes > 4 or <-4 Z-scores between 24 and 36 weeks and BMI-for-age Z-score ≥6. All the flagged anthropometric observations were assessed and values treated as missing if no plausible explanation was determined.

### Feeding pattern

We used a combination of 24-hour and 7-day infant feeding recall data at each follow-up visit to generate time specific food consumption indicator variables (for breast milk, water, sugar water, formula, cereals, fruits/vegetables, traditional herbs, prescribed and non-prescribed medicines) with 3 categories: yes, no and missing. For example if the caregiver said that she gave the child breast milk in the previous 24-hours or 7-days then we coded that child as having received breast milk. If the caregiver said “no” to both questions on breast milk, the child was then considered as one that did not receive breast milk. The response was coded as “missing” for breast milk if data were missing for both questions. Cross-tabulation of the 12 week breast milk and formula indicators revealed that all children had consumed at least one of the two foods. Based on exploratory analysis we combined two of the three combinations of these feeding indicators and this resulted in a binary ‘ever breastfed” variable with the following categories: yes (received breast milk with other solids and liquids which may include formula) and no (received formula and other liquids and solids except breast milk). The 12 week breastfeeding cessation variable was defined as no breastfeeding at the 12 week interview (based on 24-hour and 7-day recall) and no breastfeeding reported for the subsequent final 24-week interview. Only children who initiated breastfeeding by the 3 week visit were considered in this definition.

### Statistical analysis

Unlike the ordinary least squares (OLS) regression which only considers the conditional mean function, we used quantile regression which is a statistical technique that provides a more detailed analysis of the relationship between the dependent variable and its independent variables because it provides conditional regression coefficients for each quantile, [34,35]. We used univariate and multivariate simultaneous quantile regression to test whether 12 week infant feeding and 12–24 week growth velocity (adjusting for other variables) had increased effects over the upper tails of the conditional distribution of BMI-for-age Z-scores at 2 years. For this analysis we included the internal sample quantiles (70th and 90th centiles) that approximate the reference cut-offs of +2 and +3 Z-scores for BMI-for-age around 2 years. We also performed OLS regression modelling. The following variables were adjusted for in the multivariate models because of their epidemiologic or clinical importance: birth weight, maternal age, parity, maternal education, study arm and site. Although the child’s age and sex were taken into account in the BMI-for-age Z-score estimations, based on previous literature [36] we included an interaction term between the infant feeding and sex variables in initial regression models to test whether sex modifies the relationship between feeding and BMI-for-age Z-score. This interaction term was excluded from the final models as no effect measure modification was detected. Maternal age and parity were excluded from the final multivariate model because they were not significantly associated with 2-year BMI in the univariate analysis. The Breusch-Pagan / Cook-Weisberg test was used to check for heteroskedasticity and trends across the quantile regression percentiles were also tested. Continuous data are presented as mean ± SD or median (IQR) while categorical variables are presented as frequencies. We used the Student *t*-test to compare means and the Pearson chi-square test to examine associations in the cross-tabulations. Statistical tests were two-sided and performed at the 5% significance level. Kernel density functions were used to estimate the 2 year BMI-for-age Z-score distribution stratified by the 12 week overweight and breastfeeding while the two-sample Kolmogorov-Smirnov test was used to test for equality of the distribution functions.

### Ethical approval

The PROMISE-EBF trial was approved by the Regional Committees for Medical and Health Research Ethics (REK VEST) in Norway (issue number 05/8197), University of the Western Cape (research registration number 0607/8) and the South African Medical Research Council (protocol ID: ECO7-001). Informed consent was obtained from all participants.

## Results

### Study population

**Figure 1:**
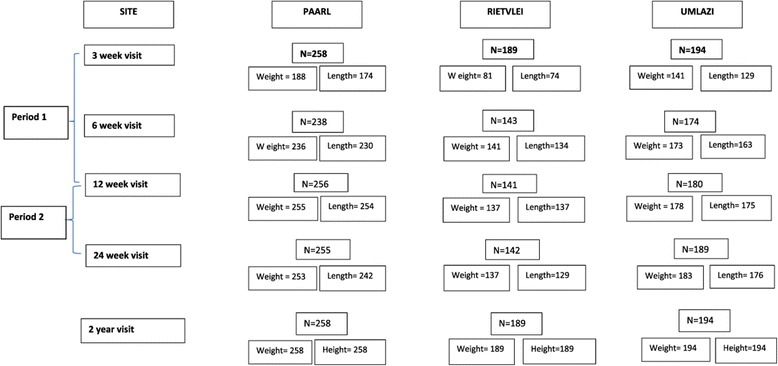
**3, 6, 12 and 24 week and 2 year weight and length data of 641 infants that were seen until the final visit.**

Six hundred and forty one HIV unexposed children with valid weight and length data (median age 22 months, IQR: 17–26 months) were analysed at the 2-year visit (Figure 1). Table 1 summarises the characteristics of these children and their mothers. Rietvlei had slightly fewer male children compared to the other sites and more children with birth weight >4 kg. About half the children were firstborn and Paarl had the highest frequency of vaginal births.

**Table 1:** **Participant characteristics by site**

**Variables**	**Paarl (N = 258)**		**Rietvlei (N = 189)**		**Umlazi (N = 194)**	
	**n**	**%**	**n**	**%**	**n**	**%**
Infant gender, (% male)	142	55.0	92	48.7	113	58.3
Birth weight, (%)						
Low birth weight (<2.5Kg)	21	8.1	6	3.2	11	5.7
Normal birth weight (2.5- < 4Kg)	219	84.9	134	70.9	172	88.7
Macrosomia (≥4Kg)	11	4.3	16	8.5	7	3.6
Missing	7	2.7	33	17.5	4	2.1
Maternal age (y), %						
15–25	160	62.0	122	64.6	134	69.1
26–44	98	37.9	67	35.5	60	30.9
Parity, %						
Primipara	126	48.8	78	41.3	114	41.2
Multipara	132	51.2	111	58.7	80	41.2
Maternal education (grade), %						
0–7	28	10.9	37	19.6	9	4.5
8–10	117	45.4	108	57.1	53	27.3
11–12	109	42.3	44	23.3	114	588
>12	4	1.6	0	0	18	9.3
Delivery mode, %						
Vaginal	217	84.1	148	78.3	116	59.8
C-section	37	14.3	26	13.8	74	38.1
Missing	4	1.6	15	7.9	4	2.1

### Infant feeding

The proportion of children consuming any breast milk decreased from 89.3% (95% confidence interval (CI), 86.5, 91.7%) at 3 weeks to 79.4% (95% CI 75.8, 82.6%) at 12 weeks (Table 2). In contrast there was an increase in the proportion of children consuming formula from 48.5% (95% CI 44.5, 52.5%) to 62.9% (95% CI 58.8, 66.9%) and cereal from 28.6% (95% CI 25.1, 32.3%) to 63.4% (95%CI 59.4, 67.4%) between the 3 and 12 weeks. Cereals were consumed by 79.5% (95% CI 71.0, 86.4%) of children who were not breastfed at 12 weeks and 59.6% (95% CI 54.9, 64.1%) of those who were breast-fed (P < 0.001). Formula was consumed by all non-breastfed children at 12 weeks and 53.7% (95% CI 49.0, 58.4%) of the breastfed children (P < 0.001).

**Table 2:** **Food consumption at 3, 12 and 24 weeks based on 24-hour and 7-day recall**

	**3 week feeding**		**12 week feeding**		**24 week feeding**	
	**(N = 629)**		**(N = 629)**		**(N = 629)**	
	**n**	**%**	**n**	**%**	**n**	**%**
Breast milk						
Yes	527	89.3*	458	79.4*	395	67.4*
Water						
Yes	269	42.8	264	45.8*	358	61.1*
Sugar water						
Yes	189	30.05	286	49.6*	154	26.3*
Traditional herbs						
Yes	424	67.4	167	28.9*	155	26.5*
Commercial infant formula						
Yes	305	48.5	363	62.9*	366	62.5*
Cereals						
Yes	180	28.6	366	63.4*	527	89.9*
Fruits/vegetables						
Yes	66	10.5	168	29.1*	400	68.3*
Prescribed medicines						
Yes	260	41.4*	231	89.5*	289	100.0*
Non-prescribed medicines						
Yes	444	70.6	355/577	61.5*	258	44.0*

*Percentage calculations were based on the total number of available data for each food item. The denominator was 629 unless otherwise indicated by the asterisk.

### Weight and length velocity

The overall mean WVZ varied between the feeding groups in both postnatal periods while no differences were observed for mean LVZ (Table 3). In period-1, children who received no breast milk at 3 weeks had a higher mean WVZ compared to children who received any breast milk (P < 0.01). The same association was observed between 12 week feeding and the period-2 mean WVZ. Comparable results were obtained when mean weight and length values were compared in place of velocity Z-scores (Additional file 2). Period-2 mean WVZ also differed by breastfeeding cessation with children who stopped all breastfeeding by 12 weeks (n = 116) having a higher (P = 0.02) mean WVZ than children who continued breastfeeding (n = 47), data not shown.

**Table 3:** **Period-1 (the 3/6 week–12 week) and period-2 (12–24 weeks) mean weight velocity (WVZ) and length velocity (LVZ) by infant feeding** ^**1**^

3 week feeding					
Period 1: 3/6–12 weeks	Never breastfed	Breastfed^3^			
	n	Mean ± SD	n	mean ± SD	P-value^2^
WVZP1 (N = 522)	60	1.58 ± 1.72	462	0.99 ± 1.60	<0.01
LVZP1 (N = 402)	46	1.69 ± 2.62	356	1.37 ± 2.44	0.41
12 week feeding					
Period 2: 12–24 weeks	Never breastfed	Breastfed^3^	P-value^2^		
WVZP2 (N = 494)	98	1.07 ± 1.75	394	0.64 ± 1.57	0.02
LVZP2 (N = 477)	93	0.82 ± 2.62	382	0.85 ± 2.52	0.93

^1^Values are mean ± SD of velocity Z-scores based on WHO standard. LVZ, length velocity Z-score; P1, Period-1; P2, Period-2; WVZ, weight velocity Z-score.

^2^Student test P values for group comparisons a 5% significance level.

^3^Children received breast milk in addition to other solids and liquids.

### Breastfed and non-breastfed children

**Figure 2:**
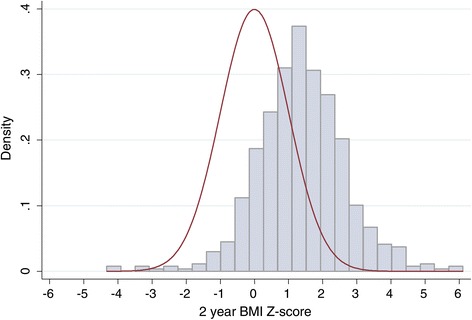
**Univariate density distribution of the sample BMI-for-age Z-scores at 2 years (blue distribution) and a normal reference distribution (red distribution).** The sample distribution is shifted to the right of the reference distribution.

**Figure 3:**
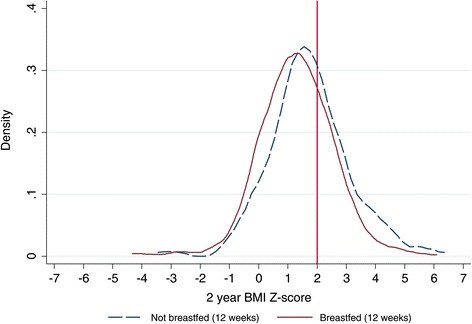
**2 year BMI-for-age Z-score estimated density distributions with regards to 12 week breastfeeding status.** The vertical red reference line indicates the +2 BMI-for-age Z-score threshold which corresponds with overweight status.

Thirty percent of all children were overweight at 2 years while 8.7% were obese. The estimated 2 year BMI-for-age Z-score density distribution for our sample is depicted in Figure 2 and is shifted to the right of the referent standard expected normal distribution. The unadjusted effect of breastfeeding status at 12 weeks (P = 0.08) on 2 year BMI-for-age Z-score is depicted in Figure 3. The mean and upper tail of the BMI-for-age Z-score density distribution for children who were not breastfed at 12 weeks are shifted to the right of the distribution for breastfed children. The adjusted linear regression showed similar results with the mean BMI-for-age Z-score at 2 years for children who were not breastfed at 12 weeks 0.32 (95% CI 0.04, 0.61) higher than for breastfed children (Table 4).

**Table 4:** **Multivariate quantile regression and ordinary least squares (OLS) coefficients for 2 year BMI-for-age Z-score** ^**1**^

	**OLS mean**	**10th percentile**	**20th percentile**	**30th percentile**	**40th percentile**	**50th percentile**	**60th percentile**	**70th percentile**	**80th percentile**	**90th percentile**
Not breastfed at 12 weeks = Yes [Ref: No]	0.32 (0.04, 0.61)	0.04 (−0.36, 0.44)	0.08 (−0.35,0.52)	0.31 (−0.10,0.72)	0.29 (−0.02,0.60)	0.37 (0.07,0.66)	0.34 (0.05,0.63)	0.46 (0.18, 0.74)	0.49 (0.16, 0.83)	0.68 (0.41, 0.94)
R-squared/pseudo R-squared	0.2182	0.1204	0.1238	0.1299	0.1251	0.1165	0.1147	0.1200	0.1216	0.1640

^1^Values are ordinary least square (OLS) and quantile regression beta-coefficients (adjusted for birth weight, weight velocity between 12–24 weeks, 12 week BMI-for-age Z-score, maternal education, study site and intervention arm) with respective 95% confidence intervals in brackets. 466 observations were assessed in the models. With the exception of study arm, only variables that had significant association with BMI-for-age Z-score in the bivariate analysis were included in the final model. R-squared/pseudo R-squared.

**Figure 4:**
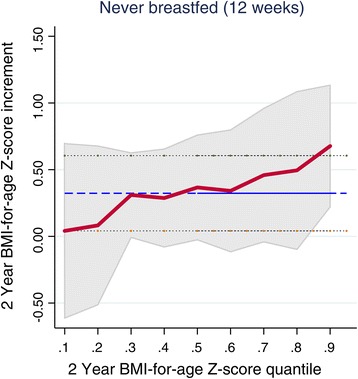
**Quantile regression and ordinary least squares (OLS) coefficients.** The figure presents the never breastfed coefficients from the final model for the 10^th^,20^th^,30^th^,40^th^, 50^th^, 60^th^,70^th^, 80^th^ and 90^th^ 2 year BMI-for-age Z-score quantiles. The respective values are connected by the maroon solid line with accompanying estimated 95% confidence interval intervals shaded in grey. The OLS value is depicted by the horizontal blue line with 95% confidence limits depicted by the black dotted lines.

Table 4 and Figure 4 present the adjusted OLS and multivariate quantile regression models (detailed table with the adjusted OLS and multivariate quantile regression models for all the covariates is in Additional file 3). The adjusted quantile regression model showed an overall significant linear trend between breastfeeding and BMI-for-age Z-score at 2 years (P = 0.01). Breastfeeding had an increasingly protective effect on the 2 year BMI-for-age Z-score conditional distribution from the 60th quantile (Table 4 and Figure 4), and this effect was most pronounced at the 90th quantile which corresponds with the definition for obesity in our sample. Children who were not breastfed at 12 weeks had a 0.37 (95%CI 0.07, 0.66) increment in BMI-for-age Z-score at the 50th quantile compared to breast-fed children. This difference in BMI-for-age Z-score increased to 0.46 (95% CI 0.18, 0.74) at the 70th quantile and was 0.49 (95% CI 0.16, 0.83) and 0.68 (95% CI 0.41, 0.94) at the 80th and 90th quantiles respectively. No significant point estimates were observed for infant feeding between the 10th and 40th quantiles. The 12 week BMI-for-age Z-score and period-2 WVZ regression coefficients showed uniform positive effects across the quantiles of 2 year BMI-for-age Z-score. The birth weight regression estimate tended to be positively associated with quantiles of the 2 year BMI-for-age Z-score and increased towards the upper tails of the distribution (Additional file 3). The assumption of homoscedasticity was not violated in the model (P = 0.52).

## Discussion

Maternal and child malnutrition, which include both under and over nutrition, are a growing concern in low and middle income countries [37]. South Africa has the highest prevalence of obesity in Sub-Saharan Africa with 42% of women, aged ≥20 years, obese and a quarter of girls younger than 20 years overweight/obese [38]. This high prevalence of obesity among women of child bearing age raises concerns as pre-pregnancy overweight/obesity and excessive gestational weight gain are risk factors for gestational diabetes, pregnancy induced hypertension [39] and adverse birth outcomes such as macrosomia. Furthermore, evidence shows that infants of obese mothers have twice the odds of being obese at 2 years compared to infants of non-obese mothers [40]. Childhood obesity is in turn a risk factor for adult obesity, diabetes and non-communicable disease [41]. To prevent this cycle of obesity, both the determinants and the critical period for overweight and obesity development must be identified.

Previous studies have identified the first 3 months of life as a critical period when infant growth is mainly nutrition dependent [14]. In this study we investigated the association between the effect of early infant feeding practices on growth velocity in the first 6 months, and the effect of 12 week infant feeding and 12–24 week growth velocity on BMI-for-age Z-score at 2 years of age amongst HIV unexposed children.

The proportion of overweight and obese children in our sample was high considering that recent findings from the first South African National Health and Nutrition Examination Survey (SANHANES-1) indicate a prevalence of overweight (BMI: 25–29.9) and obesity (BMI >30) of 17.5%(95% CI 11.4,23.6%) and 4.4%(95% CI 2.2,6.5%) respectively among boys aged 2–5 years; 18.9%(95% CI 14.1,23.4%) and 4.9%(95% CI 2.9,7.0%) among girls [42].

Consistent with results from Chirwa *et.al*. [43], our study found higher weight velocity in the first few months of life to be strongly associated with higher BMI-for-age Z-score at 2 years. We also demonstrated that children who were not breastfed at 12 weeks had higher mean WVZ between 12 and 24 weeks, higher BMI-for-age Z-scores at 2 years and were more likely to be overweight/obese. This suggests that the protective effect of breastfeeding against childhood obesity risk is partly based on its mediating effect on infant weight velocity. The higher BMI-for-age Z-scores in non-breastfed children could partly be attributed to their earlier introduction to nutrient dense cereals and formula which may have led to the rapid weight gain observed in the first 6 months of life; consistent with previous systematic reviews [44-46].

Commercial infant formulae, such as the NAN Pelargon® that was commonly used in South Africa at the time of the study, also have higher protein content (14 g/day) [47] compared to breast milk (9 g/day) [48] around 3 months. A high protein intake in excess of metabolic requirements stimulates the secretion of insulin and insulin growth factor 1 (IGF-1) axis and subsequently increases weight gain in infancy [25,49] through heightened cell proliferation and adipogenic activity (adipocyte differentiation) [50]. Insulin and IGF-1 levels are therefore generally higher in formula fed infants compared to breastfed infants [51]. Based on this “early protein hypothesis”[50] Koletzko and colleagues [25] conducted a randomized clinical trial that assessed whether growth differed between breastfed children and those fed a low protein (1.77 g/100 Kcal) formula before 6 months. Their results showed no significant difference in mean BMI and obesity risk between these two groups from 3 months of age until 6 years [25]. Nonetheless, exclusive breastfeeding continues to be strongly recommended as it has many other advantages, such as protection against gastrointestinal infections [52], which are particularly important in resource limited settings where formula feeding may not be acceptable, feasible, affordable, sustainable and safe. Breast milk also has a lower average caloric density (kcal/100 mL) [53] compared to infant formula, and the energy per kg of bodyweight in breastfed infants is 10–18% lower than for formula fed infants aged between 3 and 12 months [50]. Furthermore evidence shows that breastfed infants self-regulate the quantity and frequency of milk intake better than formula-fed infants, who tend to empty their bottles [54]. Formula-fed infants are also twice as likely to complete their meals later in infancy, even when satiated, compared to breastfed infants [55]. While cereals were given more frequently to formula-fed infants in this study, early introduction of solids was also highly practiced in breastfed infants. This early introduction of cereals could have displaced some of the breastfeeding and may therefore partially explain the higher BMI-Z-scores observed in the sample breastfed infants compared to the international reference. Previous observational studies showed that children introduced to complementary foods earlier than 3 or 4 months, compared with later introduction, were more likely to be overweight or obese [21,24,56]. Collectively, these results indicate that any breastfeeding in the first 3 months of life is protective against rapid weight gain in infancy and therefore plausibly protects against subsequent later overweight/obesity [18,36,57,58]. This further highlights the importance of early infant feeding as a key intervention to prevent childhood overweight/obesity. It is therefore of serious concern that amongst this group of HIV unexposed children, approximately half had been introduced to commercial infant formula milk by the age of three weeks. The decade of provision of free commercial infant formula milk at clinics through the Prevention of Mother to Child Transmission of HIV (PMTCT) services, together with the absence of a clear message to mothers regarding infant feeding has likely contributed to this situation [59]. Enormous efforts, detailed in the revised 2013 Infant and Young Child Feeding Policy which unambiguously supports breastfeeding as the optimal feeding mode for infants, are required to improve the EBF prevalence in South Africa [60]. Changing practices however takes time and will require collective action from all stakeholders, including communities and healthcare workers. Interventions to support breastfeeding in the workplace are also critical [61].

Our study has several strengths. To our knowledge this is the first study to assess the effect of early infant feeding on childhood BMI in African children [18,36,57,58].We are also not aware of any previous studies that have examined the effect of early weight velocity on later BMI using the WHO growth standards in this population group. We used simultaneous quantile regression, a more detailed technique than OLS regression, to examine the effect of weight velocity and infant feeding across the BMI-for-age Z-score distribution. As with Beyerlain and colleagues [62], this statistical approach enabled us to observe the differential effect of breastfeeding, which was not detected by OLS regression, on the upper tail of the conditional BMI-for-age Z-score distribution at 2 years.

Limitations of the present study were the absence of data on maternal nutritional status and smoking during pregnancy, which are known determinants of infant weight velocity and childhood overweight/obesity [63,64]. In this study we used maternal HIV status reported at recruitment; this information is subject to bias. We only included those women that reported being HIV negative in this paper; some women may have seroconverted during the follow-up period and would therefore be misclassified. The small group of HIV positive women and their children was excluded from this analysis because 1) infant HIV test results were not obtainable for one of the study sites and 2) relevant data to adjust for in this group (e.g. viral load, CD4 count and detailed information on antiretroviral (ARV) drug use) were not available. Birth weights were used to estimate the period-1 velocity for only 15 children (2.34% of the analysed sample) that did not have 3 week weight data. We therefore do not think that this small proportion of the sample skewed the results. Our feeding exposure variable was based on short term reporting of feeding practices, and not on a longitudinal assessment of feeding, at the assumed time of 12 weeks. Recall bias together with lack of precise information on the timing of the consumption of each food item and the exact quantities consumed may have therefore introduced variability in the data that is evidenced by wide confidence intervals observed in infant feeding quantile regression estimates. Furthermore, data were missing for some of the food variables because participants did not respond when asked about the consumption of that particular food item. There was also overlap between the confidence intervals of the OLS and quantile regression models and this may largely be due to the relatively modest sample size we used for this analyses approach (n = 466) compared to other studies [36,62,65].

## Conclusion

This study demonstrates that infant feeding practices in the first 12 weeks of life can predict the development of childhood overweight and obesity. Early life therefore presents a crucial window of opportunity for interventions to address modifiable factors such as infant feeding practices in order to reduce the risks of rapid weight gain in infancy and subsequent childhood overweight/obesity.

## Additional files

Additional file 1:
**Participant characteristics stratified by 2-year follow-up participation.**
Additional file 2:
**Mean weight, length/height and BMI-for-age Z-score by infant feeding** ^**1**^ **.** ^**2**^ **Student test P values for group comparisons a 5% significance level.**
Additional file 3:
**Multivariate quantile regression and ordinary least squares (OLS) coefficients for 2 year BMI-for-age Z-score.**

## Abbreviations

BMI: Body mass index
EBF: Exclusive breastfeeding
LAZ: Length-for-age Z-score
LVZ: Length velocity Z-score
PMTCT: Prevention of mother to child transmission of HIV
WVZ: Weight velocity Z-score
WAZ: Weight-for-age Z-score
WLZ: Weight-for-length Z-score
