# Correction to: Effects of early feeding on growth velocity and overweight/obesity in a cohort of HIV unexposed South African infants and children

**DOI:** 10.1186/s13006-017-0138-5

**Published:** 2017-11-13

**Authors:** Vundli Ramokolo, Carl Lombard, Meera Chhagan, Ingunn M. S. Engebretsen, Tanya Doherty, Ameena E. Goga, Lars Thore Fadnes, Wanga Zembe, Debra J. Jackson, Jan Van den Broeck

**Affiliations:** 1 Health Systems Research Unit, South African Medical Research Council, Cape Town, South Africa; 2 Centre for International Health, Department of Global Public Health and Primary Care, University of Bergen, Bergen, Norway; 3 Biostatistics Unit, South African Medical Research Council, Cape Town, South Africa; 4 School of Public Health, University of the Western Cape, Cape Town, South Africa; 5 Department of Pediatrics, University of KwaZulu Natal, KwaZulu Natal, South Africa; 6 Department of Clinical Dentistry, University of Bergen, Bergen, Norway; 7 Department of Paediatrics and Child Health, Kalafong Hospital, University of Pretoria, Pretoria, South Africa

## Correction

After publication of this article [1] it was brought to our attention that there were errors in the text under the heading ‘Data cleaning’, and in Table 3. The corrected text and updated Table 3 are given in this erratum.

**Table 3:** Period-1 (the 3/6 week–12 week) and period-2 (12–24 weeks) mean weight velocity (WVZ) and length velocity (LVZ) by infant feeding^a^

3 week feeding					
Period 1: 3/6–12 weeks	Never breastfed		Breastfed^c^		
	n	mean ± SD	n	mean ± SD	*P*-value^b^
WVZP1 (*N* = 522)	60	1.58 ± 1.72	462	0.99 ± 1.60	<0.01
LVZP1 (*N* = 402)	46	1.69 ± 2.62	356	1.37 ± 2.44	0.41
12 week feeding					
Period 2: 12–24 weeks	Never breastfed		Breastfed^c^		
	n	mean ± SD	n	mean ± SD	*P*-value^b^
WVZP2 (*N* = 494)	98	1.07 ± 1.75	394	0.64 ± 1.57	0.02
LVZP2 (*N* = 477)	93	0.82 ± 2.62	382	0.85 ± 2.52	0.93

^a^Values are mean ± SD of velocity Z-scores based on WHO standard. *LVZ* length velocity Z-score, *P1* Period, *P2* Period-2, *WVZ* weight velocity Z-score

^b^Student test *P* values for group comparisons a 5% significance level

^c^Children received breast milk in addition to other solids and liquids

“Anthropometric measurement values and Z-scores were flagged for verification if any of the following criteria were met: WAZ < −6 or >5, WLZ < −5 or >5, LAZ < −6 or >6, WLZ > 3 and LAZ < −3; 2) extreme changes in LAZ and WLZ (greater than 2.5 or 3) between consecutive visits; 3) BMI-for-age Z-score ≥ 6. All the flagged anthropometric observations were assessed and values treated as missing if no plausible explanation was determined.”
