# Supplementary material for: Effects of early feeding on growth velocity and overweight/obesity in a cohort of HIV unexposed South African infants and children
Source: Int Breastfeed J. 2015 Apr 2;10:14. doi: 10.1186/s13006-015-0041-x (PMC4396061; doi:10.1186/s13006-015-0041-x)
Supplement: Additional file 1: — Participant characteristics stratified by 2-year follow-up participation. [file 13006_2015_41_MOESM1_ESM.docx]

**Additional file 1: Participant characteristics stratified by 2-year follow-up participation**

| **Variables** | **With 2-year follow-up (N=746)** | | **Without 2-year follow-up (N=402)** | |
| --- | --- | --- | --- | --- |
| **Infant** | **n** | **%** | **n** | **%** |
| Male gender, % | 392 | 52.55 | 172 | 42.79 |
| Birth weight, % |  |  |  |  |
| Low birth weight (<2.5Kg) | 47 | 6.30 | 15 | 3.73 |
| Normal birth weight (2.5-<4Kg) | 610 | 81.77 | 326 | 81.09 |
| Macrosomia (≥4Kg) | 39 | 5.23 | 18 | 4.48 |
| Missing | 50 | 6.70 | 43 | 10.70 |
| **Maternal** |  |  |  |  |
| Age (y), % |  |  |  |  |
| 15-25 | 473 | 63.40 | 248 | 61.69 |
| 26-44 | 273 | 36.60 | 154 | 38.31 |
| Missing | 0 |  | 0 |  |
| Parity, % |  |  |  |  |
| Primipara | 391 | 52.41 | 214 | 53.23 |
| Multipara | 355 | 47.59 | 188 | 46.77 |
| Missing | 0 |  | 0 |  |
| Education (grade), % |  |  |  |  |
| 0-7 | 85 | 11.39 | 39 | 9.70 |
| 8-10 | 313 | 41.96 | 149 | 37.06 |
| 11-12 | 320 | 42.90 | 190 | 47.26 |
| >12 | 28 | 3.75 | 24 | 5.97 |
| Missing | 0 |  | 0 |  |
| Delivery mode |  |  |  |  |
| Vaginal | 545 | 73.06 | 278 | 69.15 |
| C-section | 172 | 23.06 | 92 | 22.89 |
| Missing | 29 | 3.89 | 32 | 7.96 |
