# Supplementary material for: Effects of early feeding on growth velocity and overweight/obesity in a cohort of HIV unexposed South African infants and children
Source: Int Breastfeed J. 2015 Apr 2;10:14. doi: 10.1186/s13006-015-0041-x (PMC4396061; doi:10.1186/s13006-015-0041-x)
Supplement: Additional file 2: — Mean weight, length/height and BMI-for-age Z-score by infant feeding 1 . 2 Student test P values for group comparisons a 5% significance level. [file 13006_2015_41_MOESM2_ESM.docx]

**Additional file 2. Mean weight, length/height and BMI-for-age Z-score by infant feeding^1^**

| 3 week feeding | | | | | | |
| --- | --- | --- | --- | --- | --- | --- |
|  | Never breastfed | | Breastfed^3^ | | |  |
|  | n | mean±SD | n | mean±SD | | P-value^2^ |
| Weight (Kg)  3 weeks | 43 | 4.01 ±0.75 | 347 | 4.00 ±0.62 | | 0.91 |
| 6 weeks | 55 | 4.84±0.82 | 483 | 4.93±0.79 | | 0.43 |
| Length (cm)  3 weeks | 41 | 51.69±2.34 | 336 | 51.86±2.50 | | 0.67 |
| 6 weeks | 55 | 54.69±2.76 | 472 | 55.12±2.93 | | 0.31 |
| 12 week feeding | | | | | | |
|  | Never breastfed | | Breastfed^3^ | | | P-value^2^ |
| Weight (Kg)  12 weeks | 117 | 6.32±0.89 | 451 | | 6.21±.92 | 0.25 |
| 24 weeks | 101 | 8.04±1.12 | 403 | | 7.89±1.11 | 0.22 |
| 2 years | 119 | 12.57±2.1 | 458 | | 11.62±1.84 | <0.001 |
| Length/height (cm)  12 weeks | 116 | 60.29±3.05 | 450 | | 60.03±3.05 | 0.41 |
| 24 weeks | 100 | 66.41±3.10 | 400 | | 66.49±3.49 | 0.83 |
| 2 years | 119 | 82.27±4.78 | 458 | | 80.44±4.96 | <0.001 |
| BMI-for-age Z-score  2years | 119 | 1.72±1.36 | 458 | | 1.31±1.29 | <0.01 |
|  |  |  |  | |  |  |

^1^Values are mean±SD of weight, length/height and BMI-for-age Z-score.

^2^Student test P values for group comparisons a 5% significance level.

^3^Children received breast milk in addition to other solids and liquids.
