# Supplementary material for: Effects of early feeding on growth velocity and overweight/obesity in a cohort of HIV unexposed South African infants and children
Source: Int Breastfeed J. 2015 Apr 2;10:14. doi: 10.1186/s13006-015-0041-x (PMC4396061; doi:10.1186/s13006-015-0041-x)
Supplement: Additional file 3: — Multivariate quantile regression and ordinary least squares (OLS) coefficients for 2 year BMI-for-age Z-score. [file 13006_2015_41_MOESM3_ESM.docx]

**Additional file 3: Multivariate quantile regression and ordinary least squares (OLS) coefficients for 2 year BMI-for-age Z-score ^1^**

|  | **OLS mean** | **10^th^ percentile** | **20^th^ percentile** | **30^th^ percentile** | **40^th^ percentile** | **50^th^ percentile** | **60^th^ percentile** | **70^th^ percentile** | **80^th^ percentile** | **90^th^ percentile** |
| --- | --- | --- | --- | --- | --- | --- | --- | --- | --- | --- |
| Constant | 0.26 (-0.54; 1.06) | -1.03 (-2.15;0.09 ) | -0.49 (-1.60;0.62) | -0.13 (-1.12;0.87) | 0.06 (-1.05;1.17) | 0.08 (-1.09;1.26) | 0.42 (-0.63;1.44) | 0.86 (-0.26;1.97) | 1.37 (-0.01;2.74) | 1.21 (-0.29;2.70) |
| Not breastfed at 12 weeks |  |  |  |  |  |  |  |  |  |  |
| Yes  [Ref=No] | 0.32 (0.04;0.61) | 0.04 (-0.47; 0.55) | 0.08 (-0.32;0.48 ) | 0.31 (-0.07;0.68) | 0.29 (-0.02;0.59) | 0.37 (0.09;0.64) | 0.34 (0.13;0.29) | 0.46 (0.27;0.65) | 0.49 (0.24;0.75) | 0.68 (0.31;1.03) |
| Birth weight | 0.30 (0.06;0.53) | 0.21 (-0.13;0.54) | 0.19 (-0.07;0.45) | 0.20 (-0.03;0.42) | 0.24 (-0.3;0.51) | 0.32 (0.07;0.58) | 0.29 (0.10;0.49) | 0.27 (0.03;0.51) | 0.26 (-0.10;0.62) | 0.59 (0.23;0.95) |
| WVZP2 | 0.19 (0.12;0.25) | 0.16 (0.06;0.26) | 0.21 (0.13;0.29) | 0.19 (0.12;0.26) | 0.19 (0.11;0.27) | 0.20 (0.12;0.28) | 0.20 (0.12;0.29) | 0.19 (0.10;0.28) | 0.15 (0.05;0.25) | 0.18 (0.08;0.28) |
| 12 week BMI-for-age Z-score | 0.35 (0.26;0.45) | 0.43 (0.27;0.59) | 0.40 (0.29;0.52) | 0.34 (0.23;0.44) | 0.38 (0.29;0.47) | 0.33 (0.23;0.43) | 0.34 (0.26;0.42) | 0.32 (0.24;0.40) | 0.30 (0.21;0.39) | 0.37 (0.21;0.53) |
| Education (grade) |  |  |  |  |  |  |  |  |  |  |
| 8-10 | -0.11 (-0.49;0.26) | -0.03 (-0.51;0.45) | 0.29 (-0.27;0.83) | 0.18 (-0.29;0.64) | 0.04 (-0.38;0.46) | -0.06 (-0.46;0.34) | -0.04 (-0.50;0.43) | -0.15 (-0.63;0.32) | -0.18 (-0.91;0.55) | -0.34 (-1.08;0.39) |
| 11-12 | -0.15 (-0.54;0.24) | 0.12 (-0.31;0.55) | 0.09 (-0.38;0.55) | 0.05 (-0.32;0.42) | -0.05 (-0.42;0.31) | -0.12 (-0.50;0.27) | -0.09 (-0.56;0.39) | -0.26 (-0.75;0.24) | -0.19 (-0.94;0.54) | -0.53 (-0.36;0.29) |
| >12  [Ref= 0-7] | 0.30 (-0.42;1.02) | 0.78 (0.06; 1.50) | 0.56 (-0.23;1.36) | 0.46 (-0.26;1.17) | 0.30 (-0.34;0.94) | 0.28 (-0.37;0.94) | 0.06 (-0.71;0.82) | -0.19 (-0.88;0.50) | -0.48 (-2.12;1.17) | 1.01 (-0.94;2.96) |
| Site |  |  |  |  |  |  |  |  |  |  |
| Rietvlei | -0.38 (-0.71;-0.06) | -0.35 (-0.76;0.06) | -0.30 (-0.59;-0.02) | -0.43 (-0.77;-010) | -0.25 (-0.62;0.13) | -0.19 (-0.58;0.21) | -0.16 (-0.56;0.23) | -0.23 (-0.61;0.15) | -0.34 (-0.75;0.08) | -0.72 (-1.10;0.35) |
| Umlazi  [Ref= Paarl] | -0.07 (-0.33;0.18) | -0.05 (-0.46;0.36) | -0.03 (-0.27;0.21) | -0.08 (-0.27;0.13) | -0.04 (0.28;0.21) | 0.07 (-0.16;0.30) | 0.04 (-0.14;0.21) | -0.04 (-0.30;0.22) | -0.18 (-0.45;0.09) | -0.24 (-0.63;0.16) |
| Arm |  |  |  |  |  |  |  |  |  |  |
| Intervention  [Ref= control] | 0.10 (-0.13;0.32) | 0.23 (-0.10;0.56) | 0.01 (-0.22;0.24) | 0.04 (-0.16;0.23) | -0.03 (-1.16;0.10) | 0.01 (-0.17;0.19) | 0.06 (-0.10;0.23) | 0.19 (-0.03;0.41) | 0.12 (-0.12;0.35) | -0.17 (-0.41;0.06) |
| R^2^ / Pseudo R^2^ | 0.2182 | 0.1204 | 0.1238 | 0.1299 | 0.1251 | 0.1165 | 0.1147 | 0.1200 | 0.1216 | 0.1640 |

^1^Values are ordinary least square (OLS) and quantile regression beta-coefficients with respective p-values in brackets. 466 observations were assessed in the models. With the exception of study arm, only variables that had significant association with BMIZ-score in the bivariate analysis were included in the model. WVZP2, weight velocity Z-score in period-2 (12-24 weeks
